# Inter-test reference height variability – a major error factor in Heidelberg Retina Tomography glaucoma progression analysis based on stereometric parameters changes

**Published:** 2014-09-25

**Authors:** AM Dascalu, D Stana, S Duta, IS Ardeleanu, C Savlovschi, D Serban, AP Cherecheanu

**Affiliations:** * "Carol Davila" University of Medicine and Pharmacy, Bucharest, Romania; ** University Emergency Hospital Bucharest – Ophthalmology Department; *** "Alfred Rusescu" IOMC

**Keywords:** Heidelberg retina tomography, reference height, variability, progression

## Abstract

Abstract

Purpose: To investigate the role of reference height inter-test variability upon the variability of the stereometric parameters.

Materials and method: 204 glaucomatous patients underwent a complete ophthalmological exam, including Heidelberg Retina Tomography 3 (HRT-3). The exclusion criteria were optic disc or retinal pathology that might interfere with the detection of glaucoma progression, TSD >30μm. 4 sets of data were taken during the HRT-3 exam for each patient.

Results: RH variability ranged between -198 and 187. Correlation analysis revealed a linear dependence between the inter-test variability of RH and stereometric parameters change. The most powerful correlations were observed for: RNFL Thickness (r=0.756, p<0.001), Rim Area (r=0.662, p<0.001), C/D Area Ratio (r=-0.663, p<0.001). The least correlated were Height Variation Contour (r=0.31) and Cup Shape Measure (r=0.07, p=0.3). When RH variability did not exceed 25μm, the correlations with stereometric parameters change were not statistically significant (for Rim Area, r=0.21, p>0.05, for C/D Area Ratio, r=-0.13, p=0.22, for RNFL Thickness r=0.06, p=0.52).

Conclusions: For values >25μm, the variability of the RH is a major factor determining test/retest variability for RNFL Thickness, Rim Area, C/D Area, Rim Volume and Linear C/D. Inter-test variability of RH <25μm is an important criterion for the clinical relevance of stereometric parameters changes.

## Introduction

HRT-3 software allows the early detection of the structural changes of the optic disc and peripapillary retina, based on 2 different event-based analysis algorithms: stereometric parameters change and the topographic change analysis (TCA). Based on the stereometric parameters changes on 2 or more consecutive exams, the HRT software generates the normalized changes graphics, a trend analysis. The clinical significance of the 3 ways of progression analysis is not yet fully investigated. On the other hand, not every time the 3 analysis gave concordant data. TCA seems to be the least influenced by the inter-test variability, as the results are not depending on the defining of the optic disc contour, neither on the variation of the reference height plan (a parameter which is automatically calculated by HRT software and is used in discriminating the rim and the optic cup). Both these factors influence the stereometric parameters [1-3].

 The aim of the present study was to investigate the role of the inter-test variability of the reference height (RH) upon the variability of the stereometric parameters.

## Materials and method 

204 glaucomatous patients underwent complete ophthalmological exam, computerized perimetry (Optopol PTS-910), optic disc photography and HRT3. 4 different sets of data were taken for each HRT-3 evaluation. 

 The inclusion criteria were: 

 - POAG (diagnosed according to the European Glaucoma Guidelines), with compensated IOP under topic medical treatment; 

 The exclusion criteria were: 

 - optic disc or retinal pathology that might interfere with the detection of glaucoma; 

 - TSD<30 (quality index for HRT exams); 

## Results

 Due to the including criteria, TSD inter-test variability varied between 2 and 27 in the present study. No statistically significant correlation was evidenced between TSD variability and stereometric parameters changes. Thus, if TSD is <30 for every HRT exam taken (a good image quality is achieved), the effect of TSD variability upon stereometric parameters change analysis is neglectable. 

 RH variability ranged between -198 and 187 µm. The correlation of stereometric parameters with the RH variability is strong, especially for the rim area (RA), rim volume (RV), RNFL thickness (RNFLT), C/D area ratio (C/D area), linear C/D ratio. 

 Cup shape measure (CSM) and mean cup depth are the least dependent on RH variability. Similar results are communicated also by Strouthidis. 

 The values of Pearson' correlation coefficient (r) and p (t-test) for each of the stereometric parameters change in relation to RH variability are listed in the table below:

**Table 1:**
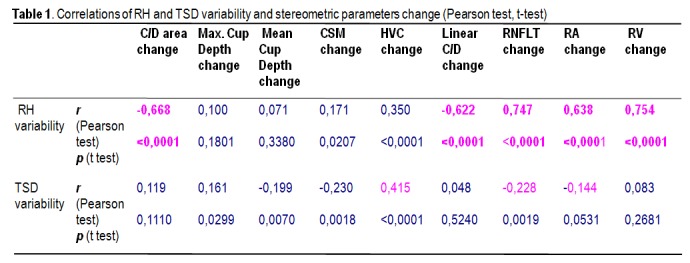
Correlations of RH and TSD variability and stereometric parameters change (Pearson test, t-test)

 Correlation analysis revealed a linear dependence between the inter-test variability of RH and stereometric parameters change. The most powerful correlations were observed for the variability (change) of: RNFL Thickness (r=0.756, p<0.001), Rim Area (r=0.662, p<0.001), C/D Area Ratio (r=-0.663, p<0.001). The least correlated were Height Variation Contour (r=0.31) and Cup Shape Measure (r=0.07, p=0.3). The increase of RH inter-test variability is directly proportional to the increase of RA, RNFLT, RV variability (change) and it is in inverse ratio to C/D area ratio and linear C/D ratio variability.

 In other words, for a bigger value of RH compared to baseline (the section taken is deeper than the baseline), HRT software calculates increased values for the following stereometric parameters: RA, RV, RNFLT, and reduced values (compared to baseline) for C/D area ratio, linear C/D ratio, cup area (CA) and cup volume (CV). In these cases, glaucoma progression may be hidden by the false increase of RA, RV and RNFLT induced by RH variation. 

 In the follow-up examinations, when RH is decreased compared to the baseline value, the reverse effect may occur: decreased values for the RA, RV, RNFLT. This situation may lead to a false result of progression of the glaucomatous defect. 

**Graph 1:**
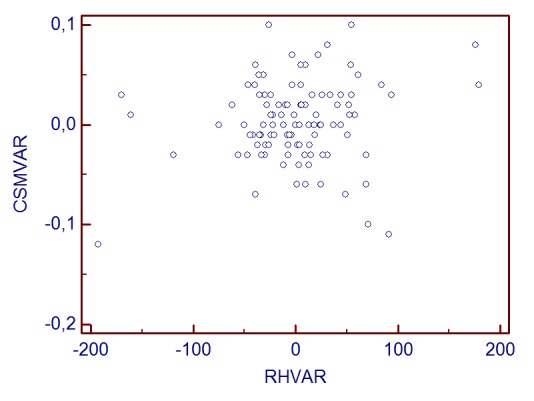
CSM change and RH variability correlation

**Graph 2:**
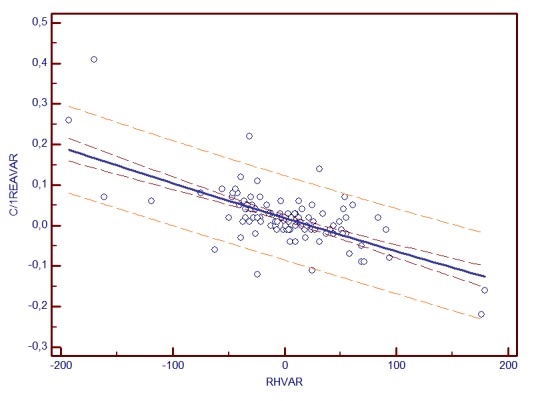
C/D AREA change and RH variability correlation

**Graph 3:**
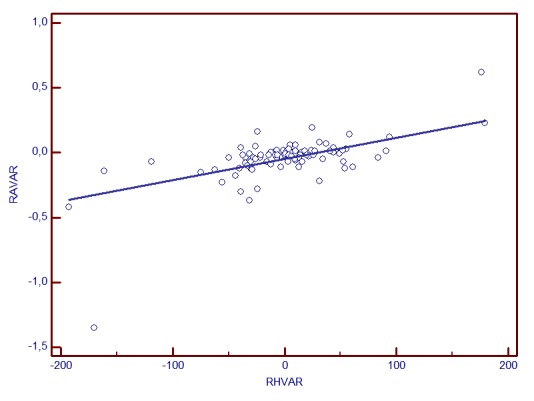
RA change and RH variability correlation

**Graph 4:**
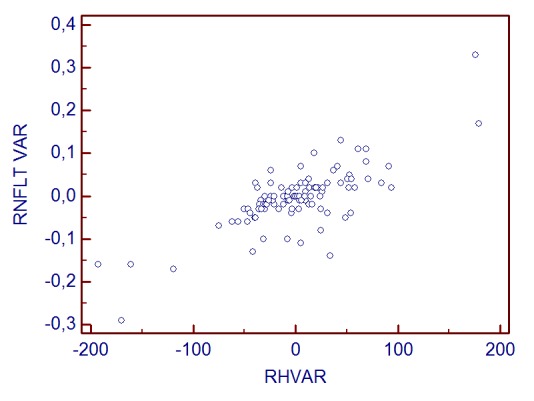
RNFLT change and RH variability correlation

 The statistical analysis showed that when RH variability did not exceed 25μm, the correlations with stereometric parameters change were not statistically significant (for fim area, r=0.21, p>0.05, for C/D Area Ratio, r=-0.13, p=0.22, for RNFL Thickness r=0.06, p=0.52).

**Graph 5:**
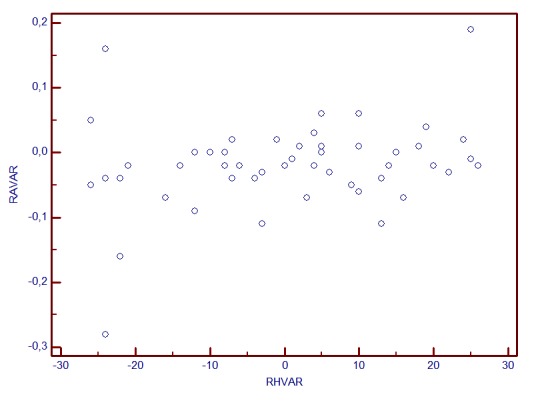
RNFLT change and RH variability correlation

**Graph 6:**
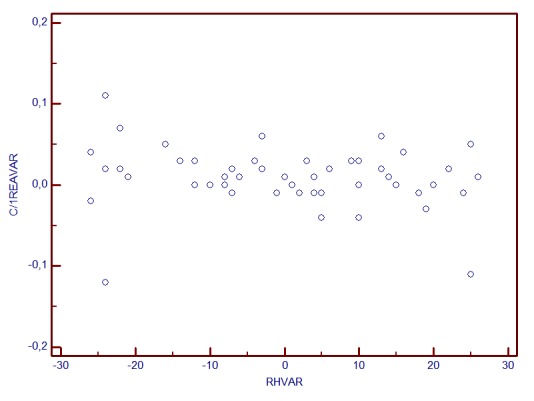
RNFLT change and RH variability correlation

**Graph 7:**
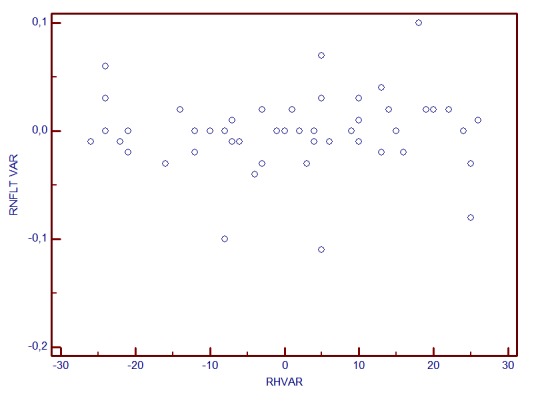
RNFLT change and RH variability correlation

 When judging between the test-retest variability and the progression of the glaucomatous defect, limited inter-test variability of RH (<25 micrometers) is an important factor to take into account. This way, any change in value of the stereometric parameters is more likely to reflect a real change in RNFLT and optic disc and not just a false result as a consequence of the various depth of the reference plan. Repeating the HRT exam in order to achieve a variability of the RH below 25 micrometers from baseline is mandatory to achieve a clinically relevant result. 

## Discussions and conclusions

 Several studies evaluated the performances of HRT-3 in terms of diagnosis of glaucomatous progression in relation to different other structural or functional investigations [**4-6**]. There are though few pieces of information on the causes of disconcordant results between topographic change analysis and stereometric parameters change and regarding the minimal criteria that must be taken into account when comparing 2 or more HRT reports of the same patient. The present study investigates the impact of the reference height variation upon the stereometric parameters. 

 In a review of literature, Topographic standard deviation – TSD [**1,7-8**] and Reference height – RH [**4,8-10**] are described as the main factors that induce inter-test variability of the stereometric parameters. Breuseggen found that in clinical practice 45,8% of the HRT-exams exhibit an increased variability of the RH, with a medium value of 29 micrometers. The impact rate upon the variability of the stereometric parameters is estimated to 55% for rim area (RA), 75% for rim volume (RV), 74% for mean RNFL thickness, 0% for cup shape measure (CSM) and just 19% for HVC. On the contrary, Strouthidis and col. [**9**] found that the rim area and CSM are the least affected by inter-test variability and recommend that the two parameters are for the early detection of glaucoma progression. 

 The present study found a significant statistical correlation between inter-test variability of RH and the following stereometric parameters: rim area, rim volume, C/D area, linear C/D ratio and RNFLT. The TSD variation of <30 proved to have no statistically significant impact upon the variability of the stereometric parameters.

 A correlated analysis of the information offered by topographic change analysis (TCA) and stereometric parameters change increased the clinical value of the HRT exams in glaucoma follow-up. Obtaining an inter-test variability of RH in ophthalmological practice, that does not exceed 25 micrometers, is essential for the clinical value of the results. Beside the limits of this confidence interval, the relevance of a change detected for the stereometric parameters is limited. 

 A message from the HRT analysis software regarding the low relevance of the results in such cases would be useful in drawing attention to the examiner to repeat the test until a variation of RH lower than 25 micrometers compared to the exam set as baseline is obtained.

 The authors have no financial interest and no proprietary interest. 

 All authors have the same contribution.
